# Inevitable Posterior Lower Segment Cesarean Section Due to Irreducible Uterine Torsion in a Woman Carrying X-Linked Myotubular Myopathy: A Case Report

**DOI:** 10.7759/cureus.95796

**Published:** 2025-10-31

**Authors:** Nouar M Elzewawi, Aseel A Almandeel, Ghada F Aldossary, Mamoun M Elawad, Khadija Z Alqahtani

**Affiliations:** 1 Obstetrics and Gynaecology, King Abdullah bin Abdulaziz University Hospital, Riyadh, SAU

**Keywords:** genetic disorder, mtm1 gene mutation, posterior lower segment cs, saudi arabia, uterine torsion, x-linked myotubular myopathy

## Abstract

We report a 38-year-old woman with secondary infertility who became pregnant by in vitro fertilization (IVF). Her pregnancy was complicated by significant and advanced polyhydramnios. At 37 weeks and five days, she was in labor with an unstable fetal condition, and an emergency cesarean section (CS) was required. The intraoperative findings showed 180° uterine torsion, which could not be reduced. A lower posterior transverse hysterectomy was successful in delivering the newborn. The male newborn exhibited severe hypotonia and respiratory failure and was subsequently diagnosed with X-linked myotubular myopathy (XLMTM) via whole exome sequencing (WES), which identified a pathogenic myotubularin 1 (MTM1) gene mutation. Maternal testing confirmed her status as an asymptomatic carrier. This case highlights the profound diagnostic and surgical challenges of uterine torsion. It posits a dual-hit model for its etiology, where fetal XLMTM caused polyhydramnios, the primary mechanical risk factor, while the maternal genetic carrier status is proposed as a novel, speculative hypothesis for a potential underlying predisposition to uterine instability. This observation requires further research to explore the possible genetic contribution to this rare obstetric emergency.

## Introduction

Uterine torsion refers to the abnormal rotation of the uterus by more than 45 degrees along its longitudinal axis, most commonly occurring in a clockwise (dextrorotatory) direction in approximately two-thirds of reported cases [1]. While a rotation of more than 45 degrees is conventionally used as the diagnostic threshold to distinguish pathological torsion from common physiological rotation, the literature varies, with some sources emphasizing rotations severe enough to cause clinical symptoms or vascular compromise. The true incidence of uterine torsion is difficult to ascertain, but it is estimated to be between one in 1,000 and one in 5,000 deliveries. Its diagnosis remains a significant clinical challenge due to the nonspecific nature of its presenting symptoms, which can mimic more common obstetric conditions. Consequently, the vast majority of cases are unexpected findings at the time of cesarean section (CS) or laparotomy, underscoring the need for heightened clinical awareness [1]. It is a rare but significant obstetric condition that has serious risks to both the mother and the fetus. It can occur in both gravid and non-gravid uterus across all reproductive age groups, although it is most observed in the third trimester of pregnancy. Uterine torsion peaks in the third trimester due to a combination of maximum uterine size and weight, creating mechanical instability, alongside hormone-induced ligament laxity that reduces anchoring support. This makes the term uterus highly mobile and prone to rotation, especially during labor or with fetal movement [2].

Uterine torsion can manifest with a wide range of nonspecific symptoms or, in some instances, be asymptomatic. The rarity of the condition, along with its many clinical manifestations, complicates preoperative diagnosis; in most cases, uterine torsion is identified intraoperatively following cesarean section (CS) or laparotomy. Ultrasound and MRI can help; however, the findings are often missed because the condition is rare and not routinely suspected. In most cases, the diagnosis remains an intraoperative surprise [3].

The novelty of this case report lies in its presentation of a potential link between irreducible uterine torsion and maternal carrier status for a myotubularin 1 (MTM1) gene mutation, a previously unreported association that may offer new insights into the pathophysiology of this rare obstetric emergency. This case report substantially contributes to the existing literature about the diagnosis and surgical management of uterine torsion during the third trimester of pregnancy. It indicates the challenges in achieving desired outcomes in this rare and unexpected condition. Also, it raises the potential relevance of underlying genetic conditions in the pathophysiology of uterine torsion. We present the case of a 38-year-old woman with secondary infertility who conceived via in vitro fertilization (IVF) and underwent an inevitable posterior lower segment cesarean section (PLSCS) due to irreducible 180-degree uterine torsion discovered intraoperatively.

## Case presentation

**Figure 1:**
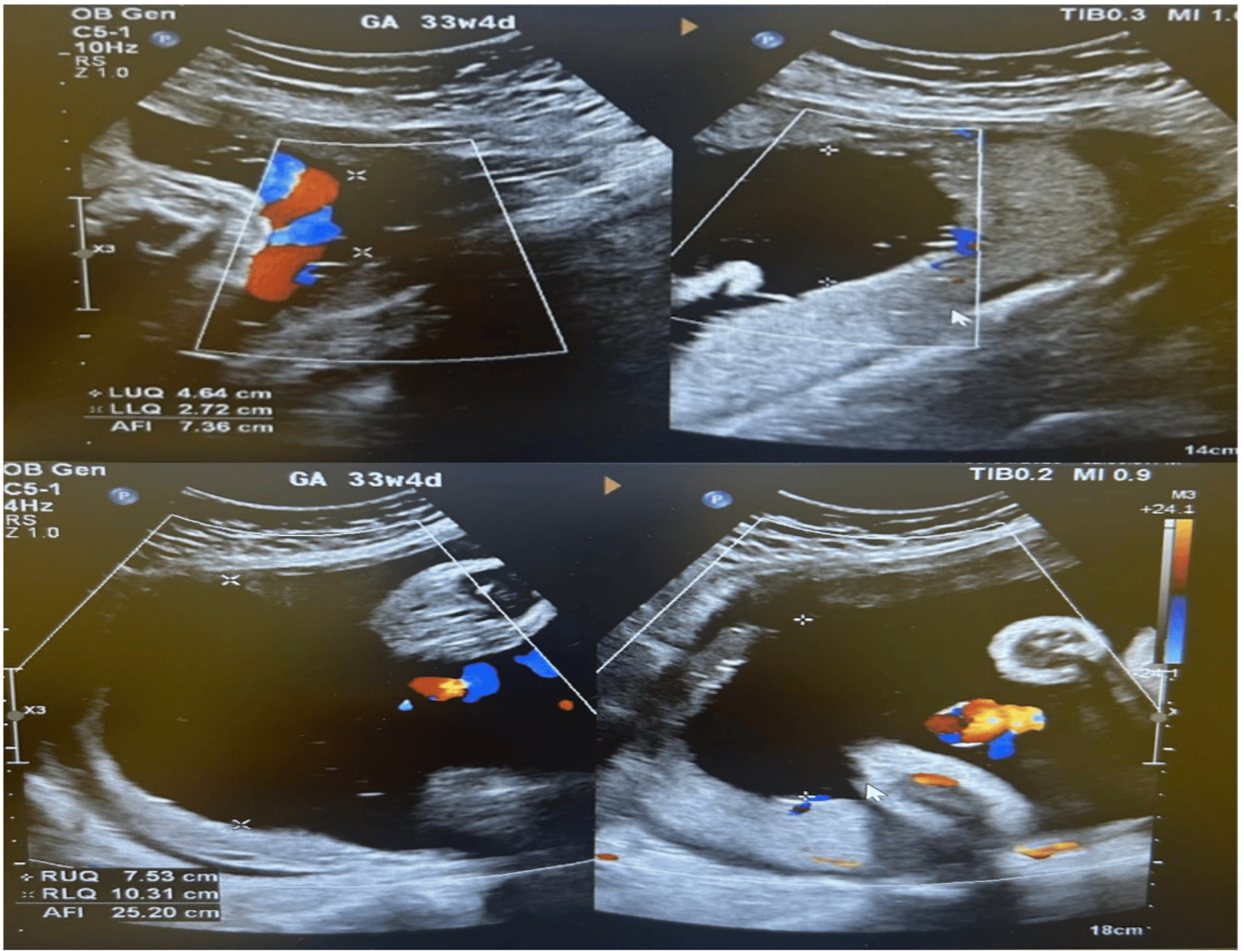
Ultrasound at 33+4 weeks demonstrating polyhydramnios (AFI: 25 cm) AFI: amniotic fluid index

We present a case of a 38-year-old Saudi woman who underwent IVF due to unexplained secondary infertility after six years of unsuccessful attempts to conceive. The patient's pre-pregnancy body mass index (BMI) was 28.4 kg/m². Her obstetric history was significant for one previous uncomplicated full-term spontaneous vaginal delivery of a healthy female infant weighing 3.2 kg. She started antenatal care at 21 weeks of gestation, during which all routine investigations, including an oral glucose tolerance test (OGTT) and an anomaly scan, returned expected results. Regular follow-up appointments were maintained throughout her pregnancy. At a gestational age (GA) of 33 weeks and four days, a routine ultrasound revealed a viable fetus with an estimated fetal weight of 2,562 g and normal growth parameters. However, polyhydramnios was noted, with an amniotic fluid index (AFI) of 25 cm, and the placenta was in the left lateral upper segment (Figure 1). The clinical data, including serial AFI measurements, fetal growth parameters, and cord pH, are now consolidated in a summary table for clarity, juxtaposed with standard reference ranges to contextualize the abnormalities. To strengthen the clinical interpretation, we have explicitly delineated the pathophysiological link: the progressive polyhydramnios (AFI > 24 cm) led to uterine overdistension, increasing rotational mobility and biomechanical instability, which culminated in the 180° dextrorotatory torsion. This structured presentation enhances the logical progression from diagnosis to management and underscores the mechanistic role of polyhydramnios in this rare complication.

**Figure 2:**
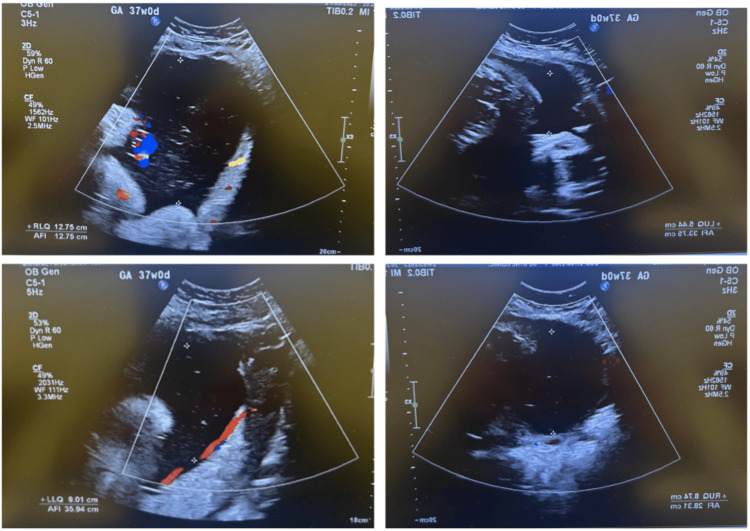
Ultrasound image showing polyhydramnios at 37 weeks Follow-up ultrasound at 37 weeks showing progression of polyhydramnios (AFI: 35.9 cm) AFI: amniotic fluid index

The patient was referred for follow-up by a maternal-fetal medicine specialist, with a subsequent scan planned for three weeks later. At 37 weeks of gestation, a follow-up ultrasound confirmed the presence of a viable fetus in a cephalic presentation, with an estimated fetal weight of 3,270 g and AFI of 35.9 cm, with normal umbilical artery Doppler results (Figure 2). A detailed follow-up ultrasound was performed to investigate the etiology of the polyhydramnios; while no structural fetal anomalies were identified, the underlying neuromuscular etiology XLMTM was not suspected prenatally in the absence of a contributory family history or definitive sonographic markers.

**Figure 3:**
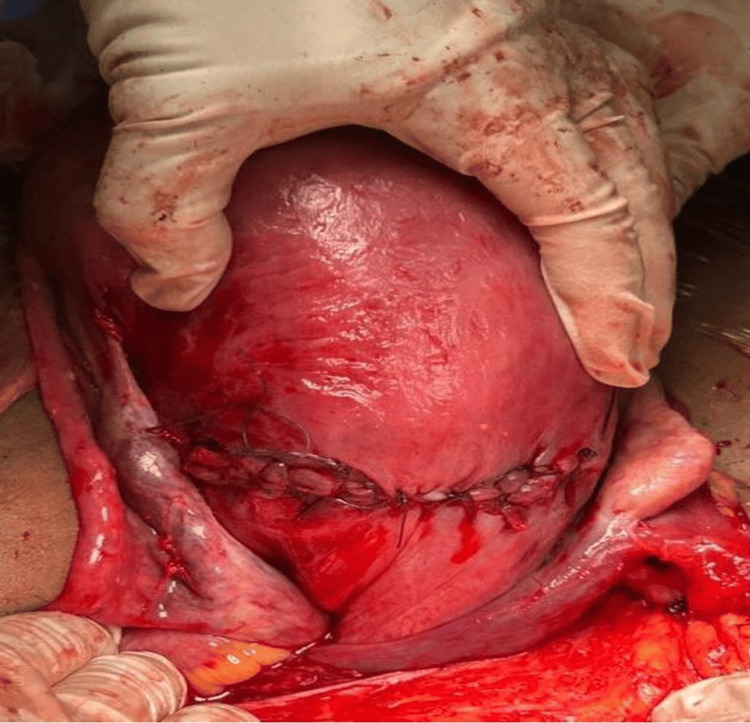
Intraoperative picture showing the torted uterus and the unavoidable incision in the posterior wall

She presented to the emergency department five days later, complaining of labor pain and a ruptured membrane. Upon assessment, vital signs were stable, the cervix was dilated to 3 cm, and the drainage was clear liquor. Due to signs of fetal distress indicated by non-reassuring cardiotocography, an emergency cesarean section was performed. During surgery, it was noted that the uterus was severely torted by 180 degrees (Figure 3). Intra-abdominal manual detorsion was attempted but failed due to uterine tenseness and immediate re-torsion. A fundal hysterotomy was considered but rejected due to concerns over its higher vascularity, significant future rupture risk, and the need for the most expeditious delivery given the non-reassuring fetal status. The posterior lower segment was the most accessible and least vascular site for a rapid transverse hysterotomy. The neonate was delivered nine minutes after the skin incision. Detorsion failed due to extreme uterine tension from polyhydramnios and fetal size, combined with severely shortened and taut uterine ligaments that immediately pulled the uterus back into its torsed position. Forcing further manipulation risked uterine rupture or vascular injury.

**Figure 4:**
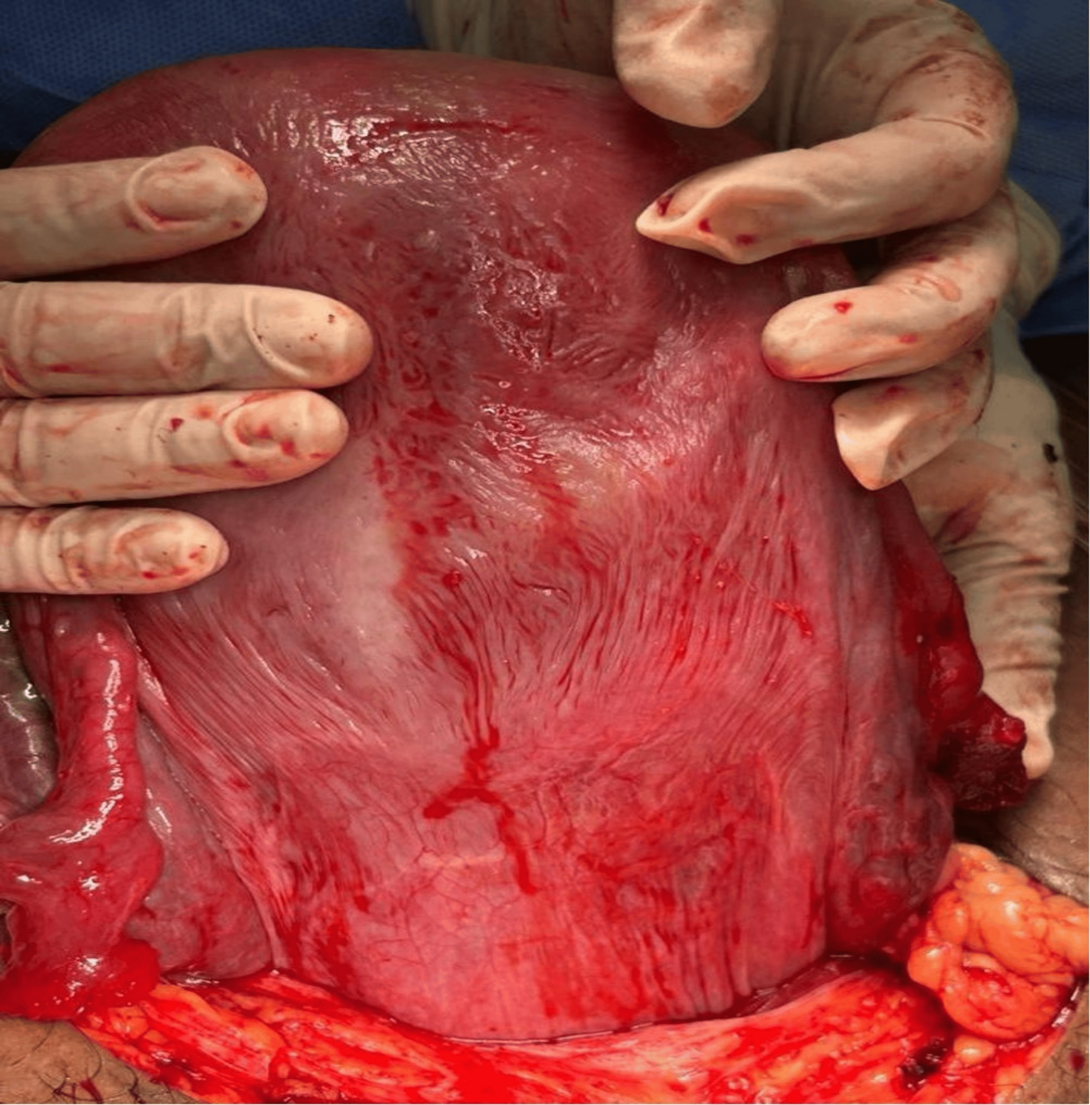
Picture taken after the correction of the position of the uterus showing the anterior wall of the uterus Posterior lower segment hysterotomy performed after failed detorsion. The anterior uterine wall is visible in the background, demonstrating the complete rotation.

Following an unsuccessful attempt at detorsion, a transverse incision at the lower segment of the posterior wall of the uterus was performed successfully to facilitate delivery of the distressed fetus (Figure 4). During surgery, the uterus was noted to be severely torted 180 degrees clockwise. Gentle manual counterclockwise detorsion was attempted but was unsuccessful due to uterine tenseness from polyhydramnios and a taut, unyielding right round ligament. As the anterior lower segment was rotated out of view and a fundal incision was considered to carry an unacceptably high risk of hemorrhage and future uterine rupture, the decision was made to proceed with a transverse incision on the accessible and less vascular posterior lower segment to expedite delivery of the compromised fetus.

The infant was a male weighing 2.9 kg, with an Apgar score of 4 at one minute and 7 at five minutes, with an arterial cord pH of 7.3. The newborn was promptly handed over to a pediatrician for immediate care. After the closure of the uterine incision, the uterus was repositioned appropriately before abdominal closure.

The patient was informed of the intraoperative findings and recovered well, being discharged 48 hours post-surgery in stable condition, and admitted to the neonatal intensive care unit (NICU) due to respiratory failure and severe hypotonia. The infant was placed on respiratory support and underwent evaluation by the neurology and genetics teams. Whole exome sequencing (WES) test showed a hemizygous pathogenic mutation in the MTM1 gene, which confirmed the diagnosis of XLMTM. Unfortunately, the infant passed away at seven months of age. The patient subsequently followed up with the genetics team, which confirmed her carrier status for the MTM1 gene mutation. The implications of X-linked recessive inheritance were discussed with her. Reduced fetal movements were not reported by the patient or documented in her antenatal records. This absence highlights a common diagnostic challenge, as fetal hypotonia from neuromuscular disorders like XLMTM may not be perceived as reduced activity by the mother.

Table 1 provides a consolidated summary of the principal quantitative findings in this case, delineating the clinical progression from antenatal presentation to definitive genetic diagnosis. The data illustrate a trajectory characterized by significant polyhydramnios with appropriate fetal growth, culminating in an acute intrapartum emergency requiring surgical intervention for irreducible uterine torsion. The subsequent postnatal genetic results confirm a hemizygous pathogenic MTM1 variant in the neonate and heterozygous carrier status in the mother, thereby establishing the underlying etiology linking the observed obstetric complications. This tabulated chronology serves to contextualize the pathophysiological sequence and facilitate cross-case comparison.

**Table 1:** Summary of key antenatal and postnatal findings Table 1 consolidates the essential clinical and genetic timeline, highlighting the progression of polyhydramnios, the occurrence of a 180° uterine torsion, and the definitive molecular diagnosis of X-linked myotubular myopathy in the neonate and carrier status in the mother. IVF: in vitro fertilization, AFI: amniotic fluid index, MTM1: myotubularin 1

Parameter	Finding	Timing/context
Maternal age	38 years	Pre-conception
Conception method	IVF	Pre-conception
AFI	25 cm	33 weeks, 4 days (initial finding)
	35.9 cm	37 weeks (progression)
Estimated fetal weight	2,562 g (~50th percentile)	33 weeks, 4 days
	3,270 g (~50th percentile)	37 weeks
Presentation	Cephalic	37 weeks scan and at delivery
Intraoperative finding	180° dextrorotatory torsion	At emergency cesarean section
Incision-to-delivery time	4 minutes	From uterine incision to neonate delivery
Neonatal arterial cord pH	7.30	At delivery
Genetic finding (neonate)	Hemizygous pathogenic variant in MTM1	Postnatal whole exome sequencing
Genetic finding (mother)	Heterozygous carrier of the MTM1 variant	Postnatal maternal testing

## Discussion

Uterine torsion is an uncommon obstetric condition characterized by vague clinical symptoms and the potential for serious maternal and fetal complications. In our case, the patient presented in labor, and the only notable preoperative finding was fetal compromise, which was later attributed to a 180-degree uterine torsion diagnosed intraoperatively. The unavoidable nine-minute incision-to-delivery interval, resulting from the irreducible torsion and unconventional posterior approach, may have contributed to the initial neonatal depression. However, the profound and refractory nature of the infant's hypotonia and respiratory failure was pathognomonic for the underlying XLMTM, with the surgical course acting as a potential aggravating factor in an already compromised neonate. Reported cases in the literature have described various presentations, including abdominal pain, vaginal bleeding, gastrointestinal disturbances, obstructed labor, hemodynamic instability, or incidental discovery during cesarean section or laparotomy [4,5]. The etiology of uterine torsion during pregnancy remains poorly understood. As the pregnancy progresses, the cervix and lower uterine segment soften and enlarge, and are associated with vascular congestion. These physiological changes, when combined with increased uterine activity, may predispose to torsion [6].

While this case raises the hypothesis of a maternal genetic predisposition, the fetal XLMTM provides a direct and sufficient pathophysiological pathway to torsion via polyhydramnios. The resulting uterine overdistension is a well-established risk factor that creates the mechanical instability necessary for such an event. The potential contribution of the maternal MTM1 carrier status to uterine biomechanics, while intriguing, remains theoretical and would require further investigation to separate it from this primary fetal mechanism.

Several structural abnormalities have been implicated as potential contributors, including uterine anomalies (e.g., bicornuate uterus or rudimentary horn), leiomyomas, adnexal masses, and sudden changes in fetal position or abnormal lie [6,7]. Additionally, Jensen performed a thorough literature review on uterine torsion cases and found numerous predisposing conditions, such as placental anomalies and uterine overdistension related to diseases [8]. Of particular interest in this case, the patient had a significant amount of polyhydramnios, which could have facilitated uterine torsion through increasing uterine size and enhancing rotational instability. An additional noteworthy aspect of this case is the postnatal diagnosis of X-linked myotubular myopathy (XLMTM) in the neonate, with subsequent genetic testing confirming the mother's carrier status for the MTM1 gene mutation. XLMTM is a rare congenital disorder caused by mutations in the MTM1 gene, affecting approximately one in 50,000 male births. It is characterized by profound hypotonia, generalized muscle weakness, delayed muscle fiber maturation, and respiratory failure. It is estimated that at least 25% of boys with severe XLMTM die in the first year of life, and those who survive rarely live into adulthood [9,10]. The potential link between maternal genetic carrier status and uterine torsion merits consideration from a biomechanical perspective. Mutations in the MTM1 gene, while primarily causing a myopathy in males, may exert subclinical effects on smooth muscle and connective tissue integrity in female carriers. This could theoretically compromise the structural support of the uterus and its ligaments, reducing its stability and predisposing it to torsion under the mechanical stress of a gravid state, particularly when compounded by factors such as polyhydramnios. This mechanism would be analogous to the increased risk of uterine torsion suggested in patients with Ehlers-Danlos syndrome (EDS), where inherent connective tissue weakness is a known factor.

Prenatal indicators often include polyhydramnios (considered the most common finding) and reduced fetal movements and, in some cases, fetal cardiac arrhythmias [10]. Most female carriers of MTM1 mutations are unaware of their status during their reproductive years, with nearly 39% first learning of the condition following the birth of a severely affected child [11]. Certain obstetric complications have been noted, such as ectopic pregnancies, placenta previa, postpartum hemorrhage due to uterine atony, urinary tract infections, preterm birth (PTB), and polyhydramnios due to an affected fetus [11]. Rudnik-Schöneborn et al. strategically published a case report on pretreatment with infliximab [11]. After all, the most robustly positive associations that have been demonstrated to date relate to infections and delivery [12], a full-term pregnancy complicated by uterine torsion in a patient with suspected Ehlers-Danlos syndrome (EDS) [13], where underlying connective tissue disorders may increase the risk of uterine torsion via decreased ligamentous support and reduced stability of the uterus [14]. The analogy between MTM1 carrier status and Ehlers-Danlos syndrome is mechanistically plausible, as both hypothesize a pathway of compromised structural tissue integrity leading to uterine instability. However, while supported by case reports for EDS, this link remains a novel and speculative hypothesis for MTM1 carriers, requiring future validation. This is consistent with the idea that a maternal carrier of certain genetic conditions, e.g., MTM1, might have pre-existing tissue laxity or altered uterine mechanics to induce torsion.

The significant polyhydramnios (AFI increasing from 25 cm to 35.9 cm) detected from 33 weeks' gestation was a pivotal finding in this case, likely serving as a key mechanical predisposing factor for uterine torsion by creating uterine overdistension and rotational instability. In retrospect, the management of this polyhydramnios warrants examination. The patient was managed expectantly, as she remained asymptomatic, with a stable cervix and no signs of preterm labor. Invasive interventions such as amnioreduction were deferred due to the associated risks of iatrogenic preterm labor, infection, and placental abruption at 33 weeks. Furthermore, pharmacological treatment with indomethacin was not initiated in the late third trimester due to concerns regarding fetal ductus arteriosus constriction. The postnatal diagnosis of XLMTM in the neonate provided the definitive explanation for the polyhydramnios, a known prenatal indicator of this condition due to impaired fetal swallowing [10]. This sequence underscores the diagnostic challenge when polyhydramnios is the sole presenting sign of an underlying fetal neuromuscular disorder and highlights the difficult risk-benefit calculus in its management, which may inadvertently permit a rare complication like uterine torsion to develop.

The management of irreducible uterine torsion presents a significant intraoperative challenge. In this case, a posterior lower segment incision was a necessary deviation from standard practice to balance fetal expediency with maternal safety, avoiding a high-risk fundal hysterotomy. While this approach facilitated delivery, the time taken for surgical decision-making and execution in this complex scenario may have contributed to the neonatal depression observed. However, the severity and nature of the infant's hypotonia and respiratory failure were ultimately indicative of the underlying XLMTM rather than the mode of delivery. Following this event, the patient's strong desire for future children was addressed with a multidisciplinary plan involving pre-conception genetic counseling and a strategy for scheduled pre-labor cesarean section in any subsequent pregnancy, given the unknown integrity of the posterior uterine scar.

## Conclusions

This case introduces a novel potential association between MTM1 carrier status and uterine torsion. While the fetal condition provided a clear mechanical pathway via polyhydramnios, a contribution from subclinical maternal myometrial dysfunction, supported by the gene's role in cytoskeletal integrity and the known risk of uterine atony in carriers, is a compelling hypothesis. This connection warrants further investigation, as it could have significant implications for the obstetric risk assessment and genetic counseling of women in known MTM1 carrier families.
